# Compensatory movement strategies differentially affect attention allocation and gait parameters in persons with Parkinson’s disease

**DOI:** 10.3389/fnhum.2022.943047

**Published:** 2022-08-18

**Authors:** Galit Yogev-Seligmann, Tal Krasovsky, Michal Kafri

**Affiliations:** ^1^Department of Occupational Therapy, Faculty of Social Welfare and Health Sciences, University of Haifa, Mount Carmel, Israel; ^2^Department of Physical Therapy, Faculty of Social Welfare and Health Sciences, University of Haifa, Mount Carmel, Israel; ^3^Department of Pediatric Rehabilitation, Sheba Medical Center, Edmond and Lily Safra Children’s Hospital, Ramat Gan, Israel

**Keywords:** Parkinson’s disease, single-channel EEG, external cues, cognitive-movement strategies, gait, attention

## Abstract

Persons with Parkinson’s disease (PwP) are advised to use compensatory strategies such as external cues or cognitive movement strategies to overcome gait disturbances. It is suggested that external cues involve the processing of sensory stimulation, while cognitive-movement strategies use attention allocation. This study aimed to compare over time changes in attention allocation in PwP between prolonged walking with cognitive movement strategy and external cues; to compare the effect of cognitive movement strategies and external cues on gait parameters; and evaluate whether these changes depend on cognitive function. Eleven PwP participated in a single-group pilot study. Participants walked for 10 min under each of three conditions: natural walking, using external cuing, using a cognitive movement strategy. Attention and gait variables were extracted from a single-channel electroencephalogram and accelerometers recordings, respectively. Attention allocation was assessed by the% of Brain Engagement Index (BEI) signals within an attentive engagement range. Cognitive function was assessed using a neuropsychological battery. The walk was divided into 2-min time segments, and the results from each 2-min segment were used to determine the effects of time and condition. Associations between cognitive function and BEI signals were tested. Findings show that in the cognitive movement strategy condition, there was a reduction in the % of BEI signals within the attentive engagement range after the first 2 min of walking. Despite this reduction the BEI did not consistently differ from natural and metronome walking. Spatiotemporal gait variables were better in the cognitive movement strategy condition relative to the other conditions. Global cognitive and information processing scores were significantly associated with the BEI only when the cognitive movement strategy was applied. In conclusion, the study shows that a cognitive movement strategy has positive effects on gait variables but may impose a higher attentional load. Furthermore, when walking using a cognitive movement strategy, persons with higher cognitive function showed elevated attentive engagement. The findings support the idea that cognitive and attentional resources are required for cognitive movement strategies in PwP. Additionally, this study provides support for using single-channel EEG to explore mechanistic aspects of clinical interventions.

## Introduction

Parkinson’s disease (PD) is associated with loss of movement automaticity due to basal ganglia dysfunction (Mirelman et al., 2019). Shifting from automatic processes to cognitive monitoring of movement offers an alternative compensatory path for movement execution (Morris et al., 2010; Abbruzzese et al., 2016). External cues—discrete targets or references for the execution of a movement—and cognitive movement strategies—focusing attention on a specific movement parameter—are common behavioral compensatory strategies in PD. Persons with PD (PwP) are advised to use these strategies to overcome typical gait disturbances (Morris et al., 2010; Spaulding et al., 2013; Rocha et al., 2014; Abbruzzese et al., 2016; Ginis et al., 2017).

Alterations in gait worsen with the progression of the disease (Mirelman et al., 2019). The alterations include changes in temporal gait variables such as reduced gait speed and a compensatory increase in the number of steps per minute (i.e., cadence) and changes in spatial gait variables such as decreased step length and arm swing. Additionally, there is an increase in gait variability and asymmetry (Hausdorff, 2009; Mirelman et al., 2019). PwP may also experience episodes of freezing of gait (FOG), which are sudden and brief episodes of inability to initiate walking or to continue moving forward (Hausdorff, 2009; Mirelman et al., 2019).

Studies and reviews of external cueing, in particular regarding temporal stimuli (e.g., rhythmic metronome beats) or spatial stimuli (e.g., spaced lines on the floor) provided evidence of a significant and immediate effect on gait variables in PwP, including step and stride length, gait speed and cadence and stride variability (Rocha et al., 2014; Ginis et al., 2018). Similarly, cognitive movement strategies, such as focusing attention on increasing step length or arm swing, were shown to improve spatiotemporal gait parameters (Morris et al., 1996; Behrman et al., 1998; Canning, 2005; Lehman et al., 2005; Baker et al., 2007; Rochester et al., 2011). Although the guidelines clearly recommend the use of these strategies, there is limited evidence to support the clinical decision process for which strategy to adopt in order to improve gait parameters. Currently, clinical decisions are based on ease of implementation, for example auditory cueing requires carrying and operating a metronome, and cognitive status of the patient. It was suggested that intact cognition is required for the use of cognitive movement strategy (Tosserams et al., 2021). To date, however, little research was done on association between cognitive profile of the PwP and effectiveness of each type of compensatory strategies. Specifically, Rochester et al. (2009) showed that PwP with cognitive decline can use cognitive movement strategy and benefit from it.

Furthermore, the underlying mechanisms of compensatory movement strategies are not well understood. It is suggested that external cues involve the processing of sensory stimulation and that cognitive movement strategies use attention allocation to and awareness of specific aspects of movement performance (Baker et al., 2007; Morris et al., 2010). Both strategies rely on cognitive resources such as attention, but it is hypothesized that cognitive movement strategies rely more strongly on cognitive resources than do external cues strategies (Weeks et al., 2001; Morris et al., 2005; Rochester et al., 2007; Morris et al., 2010).

Therefore, selection of an optimal compensatory strategy requires consideration of the cognitive deficits that are common in PwP (Litvan et al., 2011; Aarsland and Bernadotte, 2015). Reported deficits include impairments in various aspects of attention required for daily life activities, such as divided attention and sustained attention (Dubois and Pillon, 1996; Zgaljardic et al., 2003). Deficits in divided attention are extensively reported in the context of the dual-task paradigm in walking, as are their negative consequences for walking and risk of falling in PwP (Yogev-seligmann et al., 2008). Sustained attention was also reported to be impaired in PwP (Lord et al., 2010; Pfeiffer et al., 2014; Agosta et al., 2020), and this impairment was demonstrated to be associated with decrements in gait speed (Lord et al., 2010). However, despite the common recommendation for compensatory gait strategies (e.g., cognitive movement, external cueing) and the documented cognitive deficits of PwP, the attentional load of using these strategies and its association with cognitive function in PwP was not directly measured in real-life circumstances.

Previous studies showed that PwP can engage (i.e., to allocate and sustain attention) in cueing and cognitive movement strategies in a way that improves their gait (Canning, 2005; Baker et al., 2007; Rochester et al., 2011; Spaulding et al., 2013; Rocha et al., 2014; Ginis et al., 2017). Furthermore, Rochester et al. (2011) showed that PwP with cognitive decline can use cognitive movement strategy and benefit from it. However, it is not clear whether PwP can sustain their attention and engage in these strategies for prolonged periods, as is required in many cases of daily living, and whether it depends on the patient’s cognitive function.

Technology has made it possible to evaluate cognitive processes during task performance, including rehabilitation activities. Few studies have used single-channel EEG located on the frontal lobe to measure the brain engagement index (BEI), which is a measure of attention recruitment during task performance (Bartur et al., 2017, 2020; Shahaf et al., 2017). This biomarker of engagement was developed by Shahaf et al. (2017). In order to monitor subject attention, a proprietary algorithm is used to extract attention-related representations from the EEG signal. This index, which ranges from 0 to 1, marks different levels of brain attentiveness. A BEI value between 0.3 and 0.7 thresholds indicates effective engagement in the task (referred to as the “attentive engagement range” from here on). According to Gvion and Shahaf (2021), values below or above these thresholds represent affective or cognitive barriers to engagement. For example, during the odd ball task, patients with depression who did not respond to medication had lower BEI values than those who did (Shahaf et al., 2017). According to another study, young adults with attention deficit hyperactivity disorder had lower BEI values during the MOXO test, mainly below 0.3, when compared with controls (Shahaf et al., 2018).

Studies exploring the effect of these compensatory strategies used experimental settings of relatively short durations or distances (e.g., gait-analysis walkways of only several meters) (Howe et al., 2003; Hausdorff et al., 2007; Nieuwboer et al., 2007; Lohnes and Earhart, 2011), making it hard to generalize the feasibility of compensatory strategies to the ecological durations or distances required in daily living. Ginis et al. (2017) who tested the effect of different types of metronome cueing on cadence and physical fatigue during 30 min of walking, showed that participants reported fatigue yet maintained their improved cadence with different types of cueing. Although the authors explored the application of cueing for a prolonged walking time, attention during walking was not measured directly.

The general aim of the present study was to explore the motor and cognitive aspects of compensatory movement strategies in PwP during prolonged walking, and specifically: (1) to compare changes in attention allocation over time between walking with external cues or cognitive movement strategies, (2) to compare the effect of external cues and cognitive movement strategies on gait parameters, and (3) to examine the association between participants’ cognitive function and attention allocation during prolonged walking with external cues or cognitive movement strategies, in order to understand whether cognitive status is related to the ability to apply these compensatory strategies. We hypothesized that both compensatory strategies will improve gait parameters. We also hypothesized that PwP will not sustain their attention allocation during prolonged walking while using a cognitive motor strategy. Finally, we hypothesized that better cognitive function will be positively associated with the ability to allocate attention during prolonged walking.

## Materials and methods

### Participants

Participants were recruited from a community physical therapy group for PwP. They were included if they were diagnosed with PD, 50–85 years of age, able to walk independently in daily life, not using an assistive hearing device or having a hearing impairment (self-reported), not having dementia (scoring above 21 on the Montreal Cognitive Assessment (MoCA) (Nasreddine et al., 2005), and not experiencing any orthopedic condition, pain, or other health condition that might affect gait other than PD. The study was approved by the Ethics Committee of the University of Haifa. All participants provided written informed consent.

### Procedure

Participants were invited to two separate sessions to prevent fatigue. They underwent several assessments, including (1) characterization of the study population with respect to age, gender, Unified Motor Disease Rating scale (UPDRS) (Zitser et al., 2017)- Motor Examination score (part 3 of the UPDRS), Hoehn and Yahr staging (Hoehn and Yahr, 2001), disease duration, levodopa equivalent dose (LED) (Tomlinson et al., 2010) and MoCA; (2) a walking assessment including measurement of attention allocation; and (3) a computerized cognitive function assessment (Neurotrax Corp.). All participants were instructed to take their medications regularly and were in the “ON” state during the assessments. The study was conducted in a geriatric daycare center (SavYom day-care, Emek Yizrael District). Participants were recruited from May 2019 to February 2020.

### Walking conditions

Participants walked continuously on level ground for 10 min, along a 25-m corridor, under each of the following conditions: (1) natural baseline walking, in which they were instructed to walk at their natural pace (the BL condition); (2) walking using an external cue of metronome beats, with the number of beats adjusted to the cadence during usual walking (the MET condition); and (3) walking using a cognitive movement strategy in which they were instructed to focus on their step length and to take big steps (the BIG condition). BL walking was the first condition, and the remaining two conditions were randomized across participants. All participants walked in all three conditions.

### Outcome measures

#### Spatiotemporal gait variables

Gait speed, stride length, cadence, and swing time were measured continuously during each walking condition using the Mobility Lab System.^1^ Three wireless OPAL™ movement monitors (inertial measurement units, sampling rate of 128 Hz) were affixed to each participant’s legs and waist (Mobility Lab, APDM Inc., Portland, Oregon).

#### Brain engagement index

The EEG electrode was positioned on the frontal lobe (∼Fpz) (Bartur et al., 2017; Shahaf et al., 2017). The measurement was conducted using a MindWave mobile EEG headset (Neurosky™) at a sampling rate of 512 Hz. The sampled data were transferred through a wireless connection to a computer, where the signal was processed using a BrainMarc algorithm (Brain-MARC Ltd.).

#### Cognitive assessment

A computerized cognitive assessment battery (Neurotrax Corp.) was used to quantify cognitive function (Dwolatzky et al., 2003). Computerized versions of the Stroop test, Go-NoGo Response Inhibition test, Staged Information Processing Speed test, Finger Tapping test, “Catch” Game test, and Problem-Solving tests were conducted to evaluate executive function, attention, information processing speed, and motor skills (see a more detailed description in Dwolatzky et al., 2003). A composite score was calculated for each test by combining accuracy and reaction time (100 × accuracy/reaction time), taking speed-accuracy trade-offs into account. The recorded scores were used to calculate the following indices according to the manufacturer’s recommended procedures: an Executive Function index score, which measures accuracy and overall performance on executive-function-intensive tasks (e.g., Stroop Interference); an Attention index, which captures reaction times for tasks requiring a person to focus but that do not require high executive function levels; an Information Processing index, which reflects performance on low- and medium-load stages of the Staged Information Processing Speed test; and a Motor Skills index, which reflects tasks that require motor performance (Finger Tapping and the Catch game). A Global Cognitive Score (GCS) was derived by averaging these indices (Dwolatzky et al., 2003; Hausdorff et al., 2006). Validity of the battery compared with traditional measures and reliability of its summary index scores were previously reported (Dwolatzky et al., 2003).

### Data analysis

#### Brain engagement index

The BEI was developed by Brain-MARC Ltd.^2^ It is an embodiment of a normalized template (−1, + 1) matching known averaged ERP signals of attention (1,500-ms attention-related averaged ERP delta bandpass activity and the raw EEG sample (Shahaf et al., 2017). This is a common approach in advanced EEG analysis, in which a basic template is compared with a sampled signal (Shahaf et al., 2017). The BEI was computed with a moving window of 10-s segments for the period of the preceding 600 s in the BL, MET, and BIG conditions (i.e., 60 signals). Each signal was filtered in the delta bandpass normalized to the [−1, + 1] range, where −1 denoted the most negative deflection in the filtered signal and + 1 the most positive. Then, the normalized sampled signal was scanned by a moving window of the template. The averaged distance between the sampled signal and both the template and the template opposite (1 − template) were computed: If the averaged distance was less than a threshold of 0.5 from the template, the count of matches was increased, provided that no other match was found in a previous window, partly overlapping the current one; If the averaged distance exceeded the threshold, the count of no-matches was increased, provided that no other no-match was found in a previous overlapping window. The BEI is the ratio between the counts of matches and no-matches; The maximum BEI value was set to + 1, so that the BEI scale has a range of [0, 1] (Bartur et al., 2017; Shahaf et al., 2017). For data analysis, the 10 min of walking in each condition were divided into five segments of 2 min each. Within each segment, we calculated the following:% of BEI signals within the attentive engagement range (0.3–0.7),% of BEI signals < 0.3, and% of BEI signals > 0.7.

#### Gait analysis

The following gait parameters, reflecting temporal and spatial aspects of walking commonly measured in PwP, were calculated: gait speed, stride length, cadence, swing time percent. For each parameter, we created a new variable that is the average measurement of the right and left side. Similarly to the BEI, data were calculated for each 2-min segment.

#### Cognitive function

The index scores were calculated from the groups of normalized parameters that measured similar cognitive functions, as described above. Indices were normalized and fit to an IQ-like scale (mean: 100, *SD*: 15) in an age- and education-specific fashion (Hausdorff et al., 2006). The GCS was computed as the average of all index scores and served as a measure of overall cognitive battery performance (Yogev et al., 2007).

### Statistical analysis

Outcome variables were tested for normality using the Kolmogorov Smirnov test. The measurements of the gait variables did not follow a normal distribution and therefore were analyzed using non-parametric tests.

Mixed-model repeated-measures analysis of variance (RM-ANOVA) was run to examine the effect of time and condition on% of BEI in the attentive range,% of BEI above 0.7 and% of BEI below 0.3. *Post hoc* analyses were performed using Studentized Maximum Modulus (SMM). The analysis was performed using the generalized linear model GLIMIX with Gaussian distribution. The Friedman test was run to test the time and condition effects on gait variables, which had non-normal distribution. If the overall Friedman test was significant, Wilcoxon signed-rank tests were run to identify the significance between pairs of time points or conditions. In order to examine the relationships between% of BEI in the attentive range and cognitive function indices, we employed a one-way mixed-model RM-ANOVA with 1 between-subject variable (the cognitive function index) using the proc GLIMIX procedure. GLIMIX model considers the two-level hierarchical structure of the data: five measurements clustered within each participant, and fits binomial distributions of the response variable (% of BEI in attentive range) out of all signals. A *p*-value of 0.05 was considered significant; Statistical analysis was performed using SAS for Windows version 9.4 and SPSS version 27.

## Results

Eleven participants completed the full study protocol.

Table 1 presents the demographic, clinical, and cognitive score data. Participants’ mean age was 68 ± 5.42 years. Disease duration (9.18 ± 5.79), UPDRS motor part score (25.40 ± 13.55), Hoehn and Yahr staging (2.00 ± 0.44), and LED (797 ± 510.41) indicate that participants were on average at a mid-stage of the disease.

**TABLE 1:** Demographics, disease, and cognitive characteristics of the participants.

Variable	Median (25–75% range)
Age	66 (62–71)
**Disease characteristics**	
Disease duration (years)	5 (4–13)
UPDRS motor part score	22 (16–41.00)
Hoehn and Yahr stage	2.00 (2–2)
LED	600 (400–1,064)
**Cognitive tests scores**	
MoCA score	26 (24–27)
GCS	93.1 (87.6–99.4)
Executive function index score	101.2 (87–106.9)
Attention index score	98.2 (93.1–104)
Information processing index	93.1 (74.9–102.65)
Motor skills index	88.7 (84.1–98.4)

### Over time changes in attention allocation during walking with compensatory strategies

#### % of brain engagement index signals within the attentive range

**FIGURE 1:**
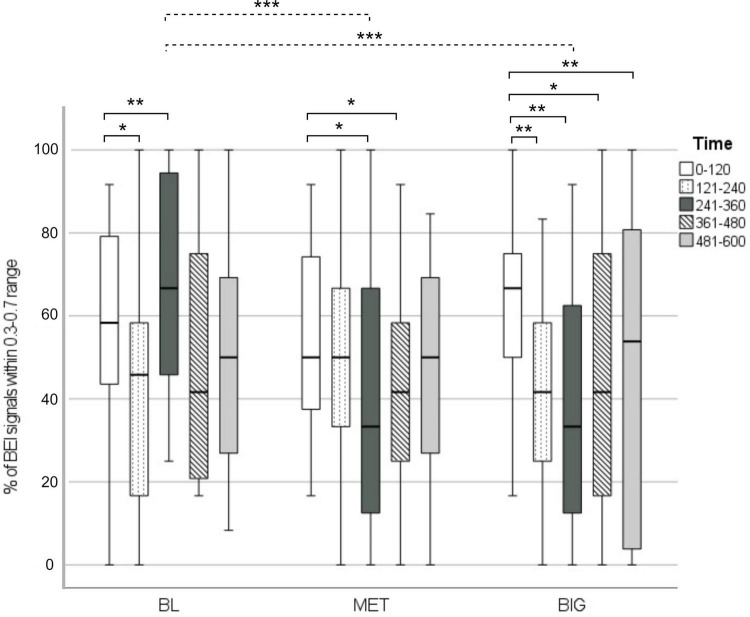
% of BEI signals within the attentive range in each walking condition, across time segments. The dashed line represents significant differences between specific time segments in walking condition. The solid lines represent within-condition differences between the first time segment and each of the subsequent segments (2–5). *p < 0.05, **p < 0.01, ***p < 0.001.

Figure 1 presents the% of BEI signals within the attentive range and the *post hoc* statistics. The% of signals within the attentive range across conditions and time segments ranged from 39 to 68%. There were significant time [*F*(4, 149) = 4.23, *p* = 0.002] and time by condition interaction [*F*(8, 149) = 3.68, *p* < 0.001] effects.

In the BL condition, BEI signals fluctuated. In both the MET and BIG conditions,% of BEI signals within the attentive range declined over time. This was more pronounced in the BIG condition, as demonstrated by the significant decline that occurred after the first 2 min and was maintained over the remaining walking duration.

#### % of brain engagement index signals above 0.7

The% of BEI signals above 0.7 across conditions and time segments ranged from 3 to 10%. BEI signals above 0.7 were observed in all time segments only during the MET condition. In the BL and BIG conditions, BEI signals above 0.7 occurred only in the second and third segment, respectively. Therefore, we ran the mixed-model RM-ANOVA only for the first two time points. There were significant time [*F*(4, 49) = 5.75, *p* = 0.02] and condition [*F*(4, 149) = 3.34, *p* = 0.002] effects, such that the% of signals above 0.7 declined over time [*t*_(49)_ = −2.58, *p* = 0.04] and was in general higher in the MET condition compared to the BIG condition [*t*_(49)_ = 2.4, *p* = 0.02].

#### % of brain engagement index signals below 0.3

The% of signals below 0.3 across conditions and time segments ranged from 33 to 57%. There was a significant time [*F*_(4, 149)_ = 4.23, *p* = 0.003] effect such that the% of signals below 0.3 increased over time. There was also a time × condition interaction [*F*_(4, 149)_ = 3.68, *p* < 0.001] effect. *Post hoc* analysis revealed that the source of the interaction was fluctuations in the BL condition.

### Effect of compensatory strategies on spatiotemporal gait variables

Table 2 presents the mean values of gait parameters in each walking condition. Participants’ gait parameters were similar to previously reported values and are indicative of mid-stage disease (Pau et al., 2018).

**TABLE 2:** Median and interquartile range of spatiotemporal gait variables.

Variable	BL walking median (25–75% range)	MET walking median (25–75% range)	BIG walking median (25–75% range)	DT walking median (25–75% range)
Gait speed (m/s)	1.08 (0.86–1.23)	1.00 (0.82–1.22)	1.05 (1.00–1.34)	0.92 (0.79–1.01)
Cadence	112.23 (109.83–117.68)	114.74 (107.60–118.28)	114.36 (103.14–118.17)	109.72 (105.41–112.01)
Swing time (% of gait cycle)	39.10 (38.06–41.63)	38.71 (38.29–41.74)	38.95 (38.54–42.77)	38.92 (37.85–41.12)
Stride length (m)	1.05 (0.95–1.27)	1.08 (0.92–1.27)	1.23 (1.11–1.35)	0.96 (0.90–1.17)

### Gait speed

Gait speed declined over time [*X*^2^ (4, *N* = 33) = 15.58, *p* = 0.004]. *Post hoc* analysis showed a decrease in gait speed in the BIG condition in all segments, comparing each segment to the first segment (Z = −2.36, *p* = 0.018; Z = −2.49, *p* = 0.013; Z = −2.1, *p* = 0.036; Z = −2.14, *p* = 0.033).

In the MET condition, gait speed decreased from the first to the second time segment (Z = −2.81, *p* = 0.005). Gait speed in all other time segments did not differ from the first segment. In the BL condition, no significant change in gait speed was recorded over the 10 min of walking.

In addition, main effects of condition were found for all time segments (segment 1: *x^2^* = 8.14, *p* = 0.017; segment 2: *x^2^* = 14.37, *p* = 0.001; segment 3: *x^2^* = 13.27, *p* = 0.001; segment 4: *x^2^* = 12.18, *p* = 0.002; and segment 5: *x^2^* = 11.49, *p* = 0.003). *Post hoc* analysis showed that the gait speed was higher in the BIG condition compared with the BL and MET conditions, for all time segments (*p*-value range 0.03–0.003), and that gait speed in the BL and MET conditions showed no significant difference.

### Stride length

Stride length significantly declined over time [*X*^2^ (4, *N* = 33) = 22.24, *p* < 0.001]. *Post hoc* analysis showed a significant decrease in stride length in the BIG condition, in all segments compared to the first segment (Z = −2.94, *p* = 0.003; Z = −2.67, *p* = 0.008; Z = −2.8, *p* = 0.005; Z = −2.67, *p* = 0.008). In the MET condition, stride length decreased in the second time segment in comparison to the first segment (Z = −2.2, *p* = 0.028), yet stride length in all other time segments did not differ from the first segment. In the BL condition, stride length showed no significant change over the 10-min walking period.

In addition, there were main effects of condition for all time segments (segment 1: *x^2^* = 16.54, *p* < 0.001; segment 2: *x^2^* = 14.47, *p* = 0.001; segment 3: *x^2^* = 14.36, *p* = 0.001; segment 4: *x^2^* = 14.36, *p* = 0.001; and segment 5: *x^2^* = 14.36, *p* = 0.001). *Post hoc* analysis showed that stride length was larger in the BIG condition in comparison to both the BL and the MET conditions, for all time segments (*p*-value range 0.003–0.006), and stride length in the BL and MET conditions showed no significant difference.

### Cadence

No main effects of time or condition were found for cadence.

### %-swing time

No main effects of time were found for % swing time. There was no main effect for the first time segment, however there were main effects of condition for time segments 2–5 (segment 2: *x^2^* = 6.54, *p* = 0.038; segment 3: *x^2^* = 6.72, *p* = 0.035; segment 4: *x^2^* = 10.36, *p* = 0.006; and segment 5: *x^2^* = 7.95, *p* = 0.019). *Post hoc* analysis showed that the% swing time was higher in the BIG condition compared to the MET condition for time segments 2–5 (*p*-value range 0.006–0.047). No difference in% swing time was found between the baseline and either the MET condition or the BIG condition.

### Associations between attention recruitment and cognitive function

In the BIG condition, significant positive associations were found between the% of BEI within the attentive range and GCS (B = 0.07, SE = 0.03, *p* = 0.04), and the Information Processing index score (B = 0.05, SE = 0.02, *p* = 0.03). In addition, we applied a similar analysis to the% of BEI signals below 0.3, since it contained a high percentage of the signals. There was a significant negative association between the% of BEI signals below 0.3 and the Information Processing index score (B = −0.05, SE = 0.02, *p* = 0.03).

## Discussion

This study aimed to compare over time changes in attention allocation in PwP between prolonged walking with cognitive movement strategy and external cues; to compare the effect of cognitive movement strategies and external cues on gait parameters; and evaluate whether these changes depend on cognitive function.

The findings of this study show that the cognitive movement strategy of directing attention to step length while walking (the BIG condition) had the most prominent effect on attention and spatiotemporal gait parameters. In the cognitive movement strategy condition, attention allocation declined after the first 2 min, however it remained similar to the level of attention allocation in the metronome or natural walking. The pattern of attention allocation while walking with a metronome was somewhat similar to the pattern observed for the cognitive movement strategy but the effect was less robust. In the baseline and external-cued walking conditions, no differences in attention allocation were found between the first and final 2 min of the walking period. Spatiotemporal gait parameters were higher while walking with cognitive movement strategy relative to the met and natural walking, despite a decline after 2 min. During the cognitive movement strategy condition, attention allocation and gait variables showed similar dynamics of change.

The BIG condition was the only condition where BEI values showed a correlation with cognitive function (GCS and Information Processing Speed indices).

### Over time changes in attention allocation during walking with compensatory strategies

The current study is the first to empirically measure attention allocation during the application of compensatory strategies in prolonged overground walking. BEI measurements indicate frontal activation of areas involving attention functions. The finding that attention allocation was more sensitive to the cognitive movement strategy relative to the others conditions, suggests that this strategy relies more on attention. These results provide evidence for the notion that application of a cognitive movement strategy poses attentional demands. Furthermore, this finding strengthens recent reports that indicated that different cortical mechanisms underlie external cueing and cognitive movement compensatory strategies (Stuart and Mancini, 2020; Tosserams et al., 2022).

Gvion and Shahaf (2021) suggested that effective engagement is represented by sustained BEI levels within the attentive index. Effective engagement may be hindered by affective responses such as anxiety or by cognitive decline, both of which impair attention allocation. Referring to these mechanisms, we can point to two main insights gained from our results. First, the reduction in BEI signals during the cognitive movement strategy may represent the intersection between limited ability to sustain attention in PwP (Zgaljardic et al., 2003; Agosta et al., 2020) and higher demands for attention imposed by the cognitive movement strategy. This intersection is less robust in the MET condition, as its attentional demands may be lower. Second, the high % of BEI values above 0.7 in the MET condition might be explained by the affective mechanism: The demand to match the steps to external rhythm may have been stressful for the participants. Gvion and Shahaf (2021) also suggested that BEI should be understood in the context of the task and its actual performance. In the current study this was enabled by the simultaneous recording of attention allocation and gait. The findings of concurrent decrease in BEI and gait performance support the previous notion of reliance on attentional resources while using cognitive movement strategies (Lehman et al., 2005; Baker et al., 2007; Morris et al., 2010). This dynamic, however, did not impair participants’ ability to maintain affective engagement, which in turn resulted in improved gait performance. In contrast, the effects of metronome use seemed to result from a different underlying mechanism, and may impose stress that disrupts efforts to effectively attend to the task. This mechanism may partially explain the absence of significant change in gait parameters in the MET condition.

It should be noted that the absence of a metronome effect on gait variables in the current study is contrary to previous evidence (Lim et al., 2005; Spaulding et al., 2013; Rocha et al., 2014). Previous reviews suggested that auditory cues increase gait speed (Lim et al., 2005; Spaulding et al., 2013; Rocha et al., 2014), cadence (Spaulding et al., 2013; Rocha et al., 2014), and stride length (Spaulding et al., 2013; Rocha et al., 2014). However, several studies reported that auditory cueing did not change these parameters (Baker et al., 2007; Lohnes and Earhart, 2011), similarly to our findings. There may be various causes for the absence of a metronome effect on gait performance. Metronome cueing, for instance, may differ in pace, based on whether it is given at a slower or a faster pace: Both applications have been shown to improve gait parameters (Willems et al., 2006). The current study used a natural cadence speed. We speculate that a higher metronome beat frequency might have had a greater effect on gait. Lack of effect may also be related to the small sample size in the current study.

Our findings offer additional insights relevant to the recent report of Tosserams et al. (2021) about the PwP’s perspective of the use of compensatory strategies. In their study, of seven studied compensatory strategies, internal cueing (i.e., cognitive movement strategies) was the second most frequently used in daily life, while external cues were the least frequently used. Additionally, they found that external cueing was reported by participants to have the lowest effect on their activity while internal cueing was in the third place out pf seven strategies. Tosserams et al. (2021) suggested that the limited use of external cueing is due to feasibility issues (such as the burden of setting the cues). Our findings empirically support patient’s reports about their perception of positive effect of the cognitive movement strategy on walking and the lack of effect of external cueing (Tosserams et al., 2021).

Cognitive function and BEI were only associated in the BIG condition. This finding supports the idea that cognitive movement strategies are sensitive to cognitive resources and facilitate normal movement through frontal networks, whereas external cueing may use other neural networks. Moreover, the nature of the associations indicates that PwP with higher global cognitive ability and higher information processing ability are more attentively engaged when walking with a cognitive movement strategy, whereas PwP with low information processing ability have increased cognitive or affective barriers to attentive engagement (i.e., a higher % of BEI signals below 0.3). Similarly, Tosserams et al. (2021) suggested that limited availability of cognitive resources may hinder the use of internal cues.

Only a few previous studies directly investigated the effect of external cues in comparison to a cognitive movement strategy on gait in PwP (Morris et al., 1996; Baker et al., 2007; Rochester et al., 2011). Our study shows the superiority of cognitive movement strategies over external cueing in improving gait speed, stride length, and swing-time percentage. These finding are in line with those of Baker et al. (2007) in demonstrating the advantage of cognitive movement strategies over external cues in improving spatiotemporal parameters. Our study strengthens the evidence of the benefit in instructing PwP to normalize their gait by focusing their attention on step size. Although less studied than external cues, this mode of compensatory strategy is useful and feasible in that it does not rely on external equipment.

Even though the BEI and gait variables dynamics show similar tendencies, the attention-related index may still have clinical value beyond its mechanistic value. A BEI measurement can provide clinicians with information about the affective and cognitive barriers to allocating attention effectively when walking with a compensatory strategy; information not available from gait variables. Therefore, by measuring the attention-related index, clinicians can tailor the intervention strategy according to each patient’s specific attentional response to the compensatory strategy. For example, when a patient demonstrates BEI values above the attentive engagement threshold (i.e., 0.7) during walking with a metronome, it is possible that the clinician would prefer to either use a different compensatory strategy or change the metronome bits frequency.

In addition, the incorporation of prolonged walking periods allowed us to provide valid ecological insights that may translate into clinical practice decision making. Commonly, in studies of compensatory strategies in PwP, walking durations are approximately 2 min and distance does not exceed tens of meters (Howe et al., 2003; Hausdorff et al., 2007; Nieuwboer et al., 2007; Lohnes and Earhart, 2011). We found that the first 2 min are not indicative of the attention allocation and gait performance measured in longer periods of walking. The dynamic of change over time suggests that future studies investigating gait in PwP should apply longer walking durations.

### Study limitations

The current sample size was small, although significant differences emerged between conditions. Study participants had relatively preserved cognition and they were independent community dwellers, which may limit the generalizability of the study findings with respect to persons experiencing greater cognitive or walking impairments. There was no qualitative assessment of the participants’ subjective experience while using the compensatory strategies. The addition of the participants’ perspective would have assisted in capturing the full benefits or challenges of either strategy.

## Conclusion

The current study demonstrates the potential of using simultaneous recordings of attention allocation and gait for understanding the interface of cognitive and motor performance. The use of a single-channel EEG system allowed to conduct such an investigation in a clinical setting and population.

Walking with cognitive movement strategy involved increased attentional load and was followed by improvements in gait performance that was sustained for 10 min. Cognitive capacity was related to the ability to effectively allocate attention when walking with cognitive movement strategy.

## Data availability statement

The raw data supporting the conclusions of this article will be made available by the authors, without undue reservation.

## Ethics statement

The studies involving human participants were reviewed and approved by Ethics Committee, Faculty of Social Welfare and Health Sciences, University of Haifa. The participants provided their written informed consent to participate in this study.

## Author contributions

GY-S and MK contributed to conception, design of study, and data collection. GY-S, MK, and TK contributed to data analysis, interpretation of results, and writing the manuscript. All authors have read and approved the manuscript.

## Footnotes

1
http://www.apdm.com/gait-and-posture/Mobility-Lab/

2
http://brainmarc.com

## Abbreviations

ANOVA: Analysis of variance
BEI: Brain Engagement Index
CV: Coefficient of variances
EEG: Electroencephalogram
ERP: Event-related potentials
GCS: Global Cognitive Score
LED: Levodopa equivalent dose
PD: Parkinson’s disease
PwP: Persons with Parkinson’s disease
SD: Standard deviation
SMM: Studentized Maximum Modulus
UPDRS: Unified Motor Disease Rating scale.
